# A Diverse Stochastic Search Algorithm for Combination Therapeutics

**DOI:** 10.1155/2014/873436

**Published:** 2014-03-12

**Authors:** Mehmet Umut Caglar, Ranadip Pal

**Affiliations:** ^1^Department of Physics, Texas Tech University, P.O. Box 41051, Lubbock, TX 79409, USA; ^2^Department of Electrical and Computer Engineering, Texas Tech University, P.O. Box 43102, Lubbock, TX 79409, USA

## Abstract

*Background*. Design of drug combination cocktails to maximize sensitivity for individual patients presents a challenge in terms of minimizing the number of experiments to attain the desired objective. The enormous number of possible drug combinations constrains exhaustive experimentation approaches, and personal variations in genetic diseases restrict the use of prior knowledge in optimization. *Results*. We present a stochastic search algorithm that consisted of a parallel experimentation phase followed by a combination of focused and diversified sequential search. We evaluated our approach on seven synthetic examples; four of them were evaluated twice with different parameters, and two biological examples of bacterial and lung cancer cell inhibition response to combination drugs. The performance of our approach as compared to recently proposed adaptive reference update approach was superior for all the examples considered, achieving an average of 45% reduction in the number of experimental iterations. *Conclusions.* As the results illustrate, the proposed diverse stochastic search algorithm can produce optimized combinations in relatively smaller number of iterative steps. This approach can be combined with available knowledge on the genetic makeup of the patient to design optimal selection of drug cocktails.

## 1. Introduction

Biological networks are complex and stochastic by nature. They are also robust and incorporate redundancy in their functionality. Thus, from the perspective of intervention, targeting individual proteins or pathways may not be sufficient for achieving a desirable outcome. For instance, solid tumors often fail to respond to monotherapy due to redundant pathways being able to carry on proliferation [1, 2]. Thus, combination therapy is often considered where multiple proteins and pathways are targeted to reduce tumor growth and avoid resistance to therapy [3, 4]. The primary concern with this approach is the enormous increase in the possible candidate concentrations that needs to be experimentally tested. One possible solution can be detailed modeling of the cellular system and design of the combination therapy based on analytical optimization and simulation. However, the kind of detailed model that captures the synergy or antagonism of drugs at different levels can require enormous amount of experimentation to infer the model parameters. Furthermore, this kind of approach may only work for molecularly targeted drugs where the specific drug targets are known, but modeling chemotherapeutic drug synergies can often be difficult. Existing approaches to drug sensitivity prediction based on genomic signatures often suffer from low accuracy [5–7]. Thus for generation of optimal drug cocktails, systematic empirical approaches tested on in-vitro patient tumor cultures are often considered. Some existing approaches include (i) systematic screening of combinations [8–10] which requires numerous test combinations, (ii) medicinal algorithmic combinatorial screen (MACS) based on laboratory drug screen for multiple drug combinations guided by sequential search using a fitness function [11], and (iii) deterministic and stochastic optimized search algorithms [12–15]. The systematic search approach should be focused on locating the global maximum instead of getting stuck in a local maximum. Furthermore, the optimization algorithm needs to be effective in search spaces, without existing prior knowledge, and easily adaptable to higher dimensional systems. Since the knowledge of the drug sensitivity distribution is unknown, the algorithm should be effective over a number of unrelated search spaces. A common problem related to the stochastic search algorithms in the literature is the normalization issue mentioned in [15]. Some of the search algorithms like Gur Game require proper normalization of the search space without having any prior information about it [13]. In order to overcome this problem, proposed algorithms should be adaptive to nonnormalized search spaces.

In this paper, we propose a diversified stochastic search algorithm (termed DSS) that does not require prior normalization of the search space and can find optimum drug concentrations efficiently. At this point, it is important to emphasize that the primary objective of the algorithm is to minimize the number of experimental steps and not the CPU time. The critical problem is the cost of the experiments that are necessary to find the most efficient combination, since the cost of biological experiments is significantly higher than the cost of computation in terms of time and money. As the number of biological experiments necessary to find the global maximum is equal to the number of steps, the objective is to minimize the experimental steps in order to reduce the cost of the overall process. With our proposed algorithm, we significantly decrease the number of steps necessary to find the maximum. The proposed iterative algorithm is based on estimating the sensitivity surface and incorporating the response of previous experimental iterations. By generating an estimate of the sensitivity distribution based on currently available response data, we are able to make larger moves in the search space as compared to smaller steps for gradient based approaches. The proposed algorithm is composed of two parts: (a) the first part consists of generating a rudimentary knowledge of the search space, (b) and in the second part, we utilize the crude knowledge of the sensitivity distribution to run a focused search to locate the exact maximums or run a diverse search to gather further information on the sensitivity distribution.

For comparing the accuracy of our proposed approach, we compare the performance of our algorithm to the recently proposed* adaptive reference update* (ARU) [15] algorithm which has been shown to outperform earlier stochastic search approaches for drug cocktail generation [13, 14]. We consider seven diverse example functions that represent possible drug interaction surfaces and also test our algorithm on two experimentally generated synergistic drug combinations. Even though our algorithm is not constrained to discretized drug levels, we have considered discretized drug concentration levels for our examples to be able to compare our results with previous studies. The results illustrate that the proposed algorithm is more efficient than the ARU algorithm for all the considered drug response surfaces. We also present a theoretical analysis of the proposed search algorithm to explain the algorithm performance.

The paper is organized as follows: the* Results* section contains the detailed performance analysis for the 9 examples, and 4 of them were analyzed for 2 different parameter sets; the* Discussion* section includes the theoretical analysis of the algorithm along with conclusions; and the search algorithm along with the surface estimation algorithm are presented in the* Methods* section.

## 2. Results

In this section, we present the performance of our proposed algorithm for nine different examples. The numbers of drugs considered in the examples are 2, 3, 4, and 5 with 21, 11, 11, and 11 discretized concentration levels, respectively, resulting in search space sizes of 21^2^, 11^3^, 11^4^, and 11^5^ for the synthetic examples and search grid sizes of 9^2^ and 10^2^ for the experimentally generated examples with two drugs and number of discretization levels of 9 and 10. As mentioned earlier, our results are compared with the latest stochastic search algorithm for drug cocktail generation (termed ARU algorithm) [15] which was shown to outperform earlier approaches [13, 14]. Similar to comparisons in [15], two parameters are primarily considered (a)* Cost*: average number of steps required to reach within 95% of the maximum sensitivity and (b)* Success Rate*: percentage of times that the search algorithm reaches 95% of the maximum sensitivity within a fixed number of steps. The details of the example functions, search parameters, and performance of our approach and ARU approach are shown in supplementary Tables 1–9 (see Supplementary Material available online at http://dx.doi.org/10.1155/2014/873436). Each table contains the problem dimensions, intervals, grid points, algorithmic parameters, and the performance comparison in terms of success percentage and average number of iterations (termed score) for our proposed approach and ARU. Two of the presented examples are based on experimentally generated data. (i) Supplementary Table 8 reports the results for bacterial (*S. aureus*) inhibition response for the drugs Trimethoprim and Sulfamethoxazole that has a synergistic effect as shown in [10]. The data surface is shown in supplementary Table 10. (ii) Supplementary Table 9 considers lung cancer inhibition response using the drugs Pentamidine and Chlorpromazine [16]. Both of these compounds have moderate antiproliferative activities on their own in-vitro in A549 lung carcinoma cells. But neither pentamidine (an antiprotozoal agent) nor chlorpromazine (an antipsychotic agent) is used clinically as a cancer drug. On the other hand, because of the synergy between them, they can prevent the growth of A549 lung carcinoma cells; in addition to that, in proper concentrations, combination is more effective than some of the commonly used cancer drugs. The data surface is shown in supplementary Table 11. The performance of our approach as compared to ARU algorithm for the nine examples is summarized in Table 1. The results indicate that we achieve 100% success rate for all nine examples (thirteen different evaluations), whereas ARU has slightly lower success rate in 2 of these examples. The primary benefit of our approach is the lower average number of iterations to reach within 95% of the maximum sensitivity. For all the examples considered, we require significantly lower average number of iterations to reach within 95% of the maximum. Note that the standard deviation of the number of iterations required to reach within 95% of the maximum is relatively small as compared to the difference in average iterations between ARU and DSS. For instance, the first example in 2 dimensions requires an average of 16 iterations for our proposed approach as compared to 46.2 iterations for ARU approach. The standard deviation (*σ*) in 100 runs of DSS algorithm is 7.99 and thus the difference in the mean runs between DSS and ARU is more than 3.7*σ*. The ARU algorithm has earlier been shown to outperform other existing algorithms such as Gurgame and its variants. Please refer to Tables 1 and 2 of [15] for the detailed comparison results of ARU with Gurgame. This strongly illustrates that the proposed algorithm is able to generate high sensitivity drug combinations in lower number of average iterations than existing approaches.

To further illustrate the significance of the proposed approach, let us consider one of the experimental example results. The experimental data on lung cancer contains the sensitivity for 102 = 100 drug concentration combinations where each drug is assumed to have one of 10 discrete concentrations. This data has been utilized to study the efficacy of the proposed algorithm. For instance, an exhaustive search approach will experimentally test the sensitivity of each of these 100 concentrations and select the one with the highest sensitivity and thus it will require 100 experimental steps. On the other hand, the stochastic search algorithms such as ARU and proposed DSS will start with random drug concentration combinations and try to sequentially select drug concentrations that will provide an improved knowledge of the sensitivity surface over these two drugs. As Table 1 shows, ARU will require an average of 12.4 sequential steps to reach a drug combination that has sensitivity within 95% of the maximum sensitivity, whereas the proposed DSS will require an average of 5.97 sequential steps to reach within 95% of the maximum sensitivity. Thus, DSS will reach within 95% of the maximum sensitivity on an average of 5.97 experimental steps, whereas ARU will require 12.4 experimental steps and exhaustive search will require 100 experimental steps. Note that since ARU and DSS are stochastic approaches, the number of sequential steps required can vary with each experimental run and the numbers 12.4 and 5.97 represent the mean of multiple experimental runs. The experimental data has been used here to provide the sensitivities for specific drug concentrations requested by the algorithms.

**Figure 1:**
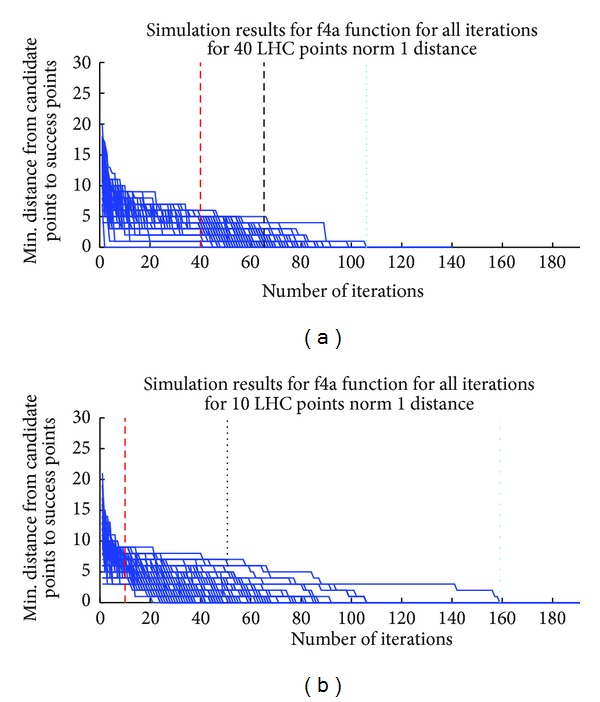
Minimum distance to optimal points for function f4a. We simulate the f4a function (see Supplementary Table 6) 100 times with two different LHC numbers. First figure represents the simulation with LHC equal to 40 and second figure represents the simulation LHC equal to 10. The analyzed function has 1 optimal point. The blue line represents the minimum norm 1 distance between the optimal points and DSS selected points. The red vertical line represents the end of Step 1, that is, Latin Hypercube Numbers, which is equal to 40th iteration in first simulation set and 10th iteration in the second simulation set; and the black vertical dotted line represents the average value of iterations (cost of proposed algorithm) required to find one of the points with ≥0.95 × *Max*⁡_efficacy_ (equal to 79.25). The cyan vertical dotted line represents the worst situation out of 100 different runs.

**Figure 2:**
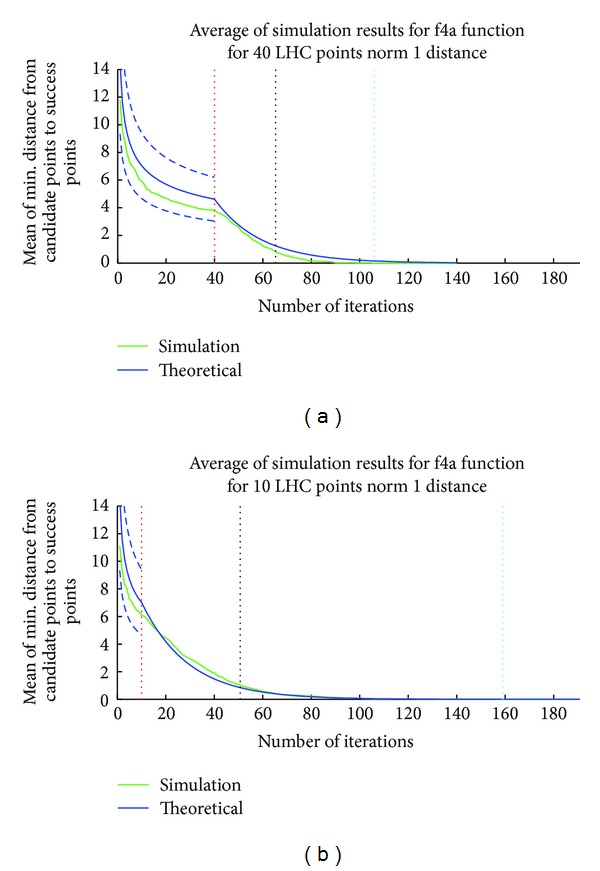
Simulation average and theoretical expected minimum distance to optimal points for function f4a (Supplementary Table 6). The blue line represents the theoretical values for *L*_1_ distance and the dashed blue lines represent the error margins (*μ* ± *σ*) for the analytically calculated values for Step 1 of the iteration. First figure represents the simulation with LHC equal to 40 and second figure represents the simulation LHC equal to 10. The red vertical line represents the end of Step 1, that is, Latin Hypercube Numbers, which is equal to 40th iteration; and the black vertical line represents the average value of iterations (cost of proposed algorithm) required to find one of the points with ≥0.95 × *Max*⁡_efficacy_. The perpendicular cyan line represents the worst situation out of 100 different runs.

For analyzing the behavior of our algorithm during the iteration process, we analyzed the minimal distance of the optimal point(s) from the DSS selected points. Let us consider *n* drugs and 0 to *T* discretization levels for each drug.  Figure 1 represents the minimum *L*_1_ distance of the points selected for experimentation to any of the optimal point(s) for the synthetic Example 6 (the simulation details are included in supplementary Table 6) with two different parameter sets (the number of initial points is 40 and 10, resp.) for 100 repeated experiments. Note that *n* = 4 and *T* = 10 for the example and thus the maximum possible *L*_1_ distance is 40. The number of optimal points for this example is 1. The red vertical line represents the value of *m* which is 40 and 10, respectively, for two different solutions of this example. The black vertical line represents the average number of iterations required to reach an optimal point for the specific response function. The cyan vertical dotted line represents the worst situation out of 100 different runs. The average minimum distance of the experimental points to the optimal point is shown in green in Figure 2. The solid blue line represents the analytically calculated expected minimum *L*_1_ distance. The theoretical analysis of the minimum distance is included in Section 3. Note that there is a change in the shape of the blue curve after the end of Step 1 (iteration 40 for Figure 2(a) and iteration 10 for Figure 2(b)). The dotted blue curve denotes the analytically calculated *μ* ± *σ* where *μ* and *σ* denote the mean and standard deviation for the minimum distance. Figures 1 and 2 illustrate that the minimum distance of the selected points to the optimal points decreases with successive iteration and closely matches the analytical predictions.

## 3. Discussion

In this section, we provide a generalized analysis of the proposed search algorithm followed by conclusions and future research directions.

### 3.1. Theoretical Analysis

In this subsection, we will attempt to theoretically analyze the distance of the point with the global sensitivity maximum from the points that are tested by the proposed algorithm. We will consider that each drug is discretized from 0 to *T* levels and that we are considering *n* drugs. Thus, any drug cocktail can be represented by a *n* length vector *V* = {*V*(1), *V*(2),…, *V*(*n*)}, where *V*(*i*) ∈ {0,1, 2,…, *T*} for *i* ∈ {0,1,…, *n*}. Thus, the search space of drug cocktails (denoted by *Ω*) is of size (*T* + 1)^*n*^ and represents points in an *n*-dimensional hypercube of length *T*. Let *V*_max⁡_ denote the drug cocktail with the maximum sensitivity among the (*T* + 1)^*n*^ points. The mapping from the drug cocktail to sensitivity will be denoted by the function *f* : *Ω* → [0 1]; that is, the maximum sensitivity will be given by *f*(*V*_max⁡_). We will assume that if the distance of the test point (*V*) from the point with the global maximum (*V*_max⁡_) is small, the sensitivity will be close to the global maximum; that is, a small |*V*_max⁡_ − *V*| will imply a small |*f*(*V*_max⁡_) − *f*(*V*)|. We will primarily analyze the *L*_1_ norm of |*V*_max⁡_ − *V*|.

Note that |*V*_max⁡_ − *V*|_1_ = ∑_*i*=1_^*n*^|*V*_max⁡_(*i*) − *V*(*i*)|. The first *m* points in our algorithm are chosen randomly in the search space and thus we will consider that *V*(*i*) has a uniform distribution between 0 and *T*. *V*_max⁡_ can also be situated in any portion of the search space and thus we will consider *V*_max⁡_ to have a uniform distribution between 0 and *T*. Thus, the probability mass function of the random variable *Z* = *V*(*i*) − *V*_max⁡_(*i*) will be given by

(1) $$\begin{array}{c} f_{Z}\left(z\right)=\{\begin{array}{cc} \frac{T+1-\left|z\right|}{{\left(T+1\right)}^{2}} & z=\left\{-T,-T+1,\ldots ,T-1,T\right\}\\ 0 & \text{otherwise}. \end{array} \end{array}$$

Subsequently, the PMF of the random variable *W* = |*Z*| will be given by

(2) $$\begin{array}{c} f_{W}\left(w\right)=\{\begin{array}{cc} \frac{2\left(T+1-w\right)}{{\left(T+1\right)}^{2}} & w=\left\{0,\ldots ,T-1,T\right\}\\ \frac{1}{T+1} & w=0\\ 0 & \text{otherwise}. \end{array} \end{array}$$

**Figure 3:**
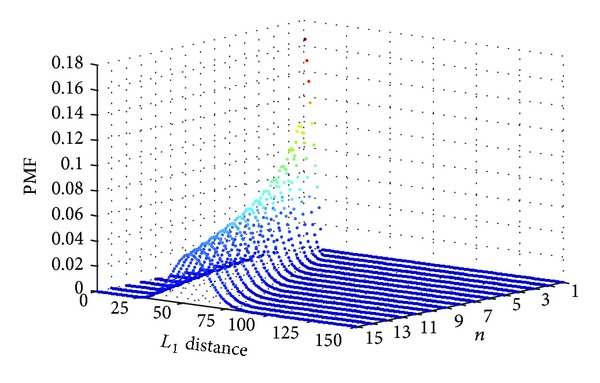
Distribution of random variable *R*_1_ (denoting *L*_1_ distance from optimal point) for *T* = 10 and different values of *n*.

The random variable *R*_1_ denoting the *L*_1_ norm |*V*_max⁡_ − *V*|_1_ = ∑_*i*=1_^*n*^|*V*_max⁡_(*i*) − *V*(*i*)| will be a sum of *n* random variables with PMF given by (2). The distribution for the sum of any two random variables consists of the convolution of the individual distributions of the random variables. Thus, the probability distribution of *R*_1_ can be calculated by convolving *f*_*W*_ *n* times. The distribution of *R*_1_ for *T* = 10 and *n* = {1,…, 15} is shown in Figure 3.

At the beginning of our algorithm, we are selecting *m* points in random. Thus, the nearest neighbor distance from the optimal point will be given by the random variable *R*_2_ that denotes the minimum of *m* random variables *X*_1_, *X*_2_,…, *X*_*m*_ selected independently based on the distribution of *R*_1_. Thus, the cumulative distribution function (CDF) of *R*_2_ is given by [17]

(3) P(R2≤x)=1−P(X1>x,…,Xm>x)=1−P(X1>x)∗⋯∗P(Xm>x)=1−(1−CDFR1(x))m.

**Figure 4:**
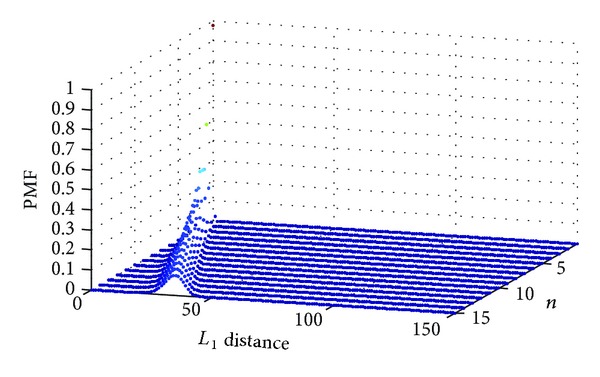
Distribution of random variable *R*_2_ (denoting the minimal *L*_1_ distance from the optimal point for *m* = 40) for *T* = 10 and different values of *n*.

The PMF of *R*_2_ given by PMF_*R*_2__(*x*) = CDF_*R*_2__(*x*) − CDF_*R*_2__(*x* − 1) for *i* = 1,2,…*nT* and PMF_*R*_2__(0) = CDF_*R*_2__(0) is shown in Figure 4.

For example, the expected minimum distance from the optimal point for *m* = 40, *T* = 10 is 6.86 for *n* = 5. The mean *μ*(*n*, *T*, *m*) and variance *σ*(*n*, *T*, *m*)^2^ of the minimum distance from the optimal point for different values of *n*, *T*, and *m* are shown in Table 2. Note that if there are *k* optimal points in diverse locations, the mean *μ*_*k*_(*n*, *T*, *m*) and variance *σ*_*k*_(*n*, *T*, *m*)^2^ of the minimum distance of the selected points from any of the optimal points are given by *μ*_*k*_(*n*, *T*, *m*) = *μ*(*n*, *T*, *k*∗*m*) and *σ*_*k*_(*n*, *T*, *m*)^2^ = *σ*(*n*, *T*, *k*∗*m*)^2^. This is because when there are *k* optimal points, the minimum distance will consist of the minimum of *m* × *k* distances (*m* distances from each optimal point). If there are multiple optimal points in one hill with small distances between each other, they will be considered as one single optimal point for the minimum distance analysis.

As Table 2 shows, the *L*_1_ distance will increase with increasing *n* and *T* and following Step 1 of the algorithm, our point with highest experimental sensitivity may not be close to the optimal point but rather may belong to another hill with a local optima. However, based on the nearest neighbor *L*_1_ distances, we would expect to have at least one point close to the optimal point in the top *k* optimal points. Thus, if we keep selecting *ρ* points for further experimentation from around the top *k* experimental points sequentially, we expect that on an average *ρ*_1_ = *ρ*/*k* points will be selected around the optimal point.

Consider that the *L*_1_ distance from the optimal point was given by the random variable *R*_2_ and if a point is selected randomly between the experimental point and the randomly selected point, the subsequent nearest neighbor distance from the optimal point will be given by the random variable *R*_3_ = *R*_2_∗*G*_1_, where *G*_1_ is a uniform random variable between 0 and 1. The distance in each dimension will be reduced by a number selected based on a uniform random variable, and consequently, we will approximate the *L*_1_ distance (sum of *n* such distances) to be reduced by a number selected based on a uniform random variable. Thus, after *ρ*_1_ points have been selected sequentially around the optimal point, the distance to the optimal point will be given by the random variable *R*_*ρ*_1__ = *R*_2_∗*G*_1_∗*G*_2_∗ ⋯ ∗*G*_*ρ*_1__. The probability distribution function of the multiplication of *ρ*_1_ random variables with uniform distribution between [0,1] is given by [18]

(4) $$\begin{array}{c} f_{G_{1}*\cdots *G_{\rho_{1}}}\left(x\right)=\frac{{\left(\ln\left(1/x\right)\right)}^{n-1}}{\left(n-1\right)!}. \end{array}$$

Thus, if the expected distance from the optimal point after the initial selection of *m* points is *D* and we select *ρ*_1_ points sequentially between the optimal point and its current nearest neighbor, the expected nearest neighbor distance from the optimal point will be *D*/2^*ρ*_1_^.

As an example, if *n* = 10, *T* = 10, and *m* = 40, we have *D* = 19.7 from Table 2. The expected *L*_1_ distance from the optimal point at the end of 40 + 20 = 60 iterative steps will be 19.7/2^6^ = 0.3078 assuming a single hill and a 0.3 probability for the focused search (path *a* of Step 2 of the algorithm). Based on the focused search probability, at the end of 60 iterations, we expect to have (60 − 40)∗0.3 = 6 points selected around the optimal point.

### 3.2. Conclusions

In this paper, we proposed a diverse stochastic search (DSS) algorithm that consisted of a parallel and sequential phase that outperformed existing efficient algorithms for drug cocktail generations on nine different examples (thirteen different evaluations). Our results show that the DSS algorithm is more efficient than the previous algorithms in terms of decreasing the number of experiments required to generate the optimum drug combination (i.e., cost of the algorithm) which in turn reduces the total cost of the drug combination search process in terms of time and money. Note that the primary costs in each sequential biological experimental step are in personnel effort to prepare the drug combination and the time involved to generate the combination drug response and thus the goal is to reduce the number of sequential steps. One of the limitations of the current approach is the computational complexity when the number of drugs (*n*) is large. The proposed method is suitable for selection of optimal drug concentrations when the number of candidate drugs has been reduced from hundreds to around ten. A number of approaches can be applied to achieve the selection of candidate drugs. For instance, an application of a drug screen to measure cell viability of the tumor culture can be utilized to narrow down the drugs to be included in a combination drug cocktail [19, 20]. Note that algorithms [19, 20] are convenient for selecting the small set of drugs for combination therapy but not for deciding on the optimal drug concentrations of the selected drugs. For possible clinical application, available genetic information can be utilized to narrow down the possible drugs to be tested and the proposed DSS algorithm can be applied to tumor cell cultures to generate the optimal concentrations of the drugs. In this paper, we also presented a theoretical analysis of the search based on minimum distance between the optimal point and the DSS selected points. Future research directions will consider incorporating the cost of drug application in the optimization process and the effect of data extraction noise on the search algorithm. The cost will be a measure of toxicity or side effects of the drug combination. One approach to incorporate the cost can consist of changing the sensitivity surface by negating the cost from the sensitivity. The cost can be simplified to be proportional to the linear addition of the individual drug concentrations. Another approach for incorporating the cost entails restricting the search space by limiting the search to areas that have cost lower than a toxicity threshold.

## 4. Methods

In this section, we present the search algorithm along with the surface mapping algorithm based on currently available information. Finally, we discuss the reasoning behind the selection of the algorithm related parameters.

### 4.1. The Search Algorithm

The primary objective of the search algorithm is to locate the global maximum in minimum number of iteration steps. Numerous approaches can be considered for this purpose and our proposed method is based on a combination of stochastic and deterministic approaches. We expect that the efficiency in terms of average number of iteration steps can be increased by large jumps over the search space rather than using traditional step-by-step gradient descent approach. Our algorithm consists of two parts: an initial parallel part and a subsequent iterative segment. The objective of the initial part is to generate a rudimentary idea of the search space. The objective of the iteration part is twofold: (a) it tries to find the exact maximum using the currently available knowledge and (b) it searches the space further to add new knowledge, that is, it attempts to find new hills that the previous iterations could not locate.

Step-by-step schema of the search algorithm is described as follows.

**Step 1**

Generation of Latin Hypercube Numbers (LHNs)

1. In this step, *m* points in the given grid are selected for drug response experiments. We first generate *m* points in the continuous search space based on LHN generation approach with the criterion of maximizing the minimum distance between these points. This approach assists in distributing the points homogeneously in the search space such that the maximum possible distance between a given target point and the nearest point whose coordinates are represented by LHN will be minimum. Consequently, we map these points to the nearest grid points and term these mapped points as approximate Latin hypercube points. We considered this continuous-discrete grid mapping to compare our results with the previous studies that utilized a grid structure for the search space.
2. In this step, experiments are conducted to determine the efficiency of the *m* drug combinations determined by the approximate Latin hypercube points.

**Step 2**

Iterative Segment

1. Normalize the experimental drug efficacy results to numbers between 0 and 1. Then the (*n* − 1)th power of the normalized drug efficacies are considered where *n* denotes the number of drugs. The power step emphasizes the hills of the distribution and the value *n* − 1 is termed as* Power used for the inputs*.
2. Estimate the drug efficacies of the unknown grid points using the sensitivity surface estimation algorithm. The details of the surface estimation algorithm are explained in subsequent sections. At the end of this procedure, we have estimates for the efficacies of each and every point on the search grid. The grid points are classified into two groups: known points from experimental data and estimated points based on interpolation and extrapolation.
3. Decide the objective of the iteration step based on a probabilistic approach. For our case, the algorithm follows* path a* to find the exact maximum based on previous knowledge with a 0.3 probability and follows* path b* with a 0.7 probability to explore the search space with a diversified approach.
4. *Path a* (Focused Search)
  - The main idea of the focused search is to experimentally search the estimated maximums generated following the surface estimation mapping. The algorithm also tries to avoid focusing on an individual local maximum by exploring geographically apart multiple estimated local maximums. To achieve this purpose, we employ a tracking algorithm to label the local maximums and avoid prolonged emphasis on individual maximum points. The individual steps of the Focused Search part are described as follows.
  - Sort the grid sensitivities (both experimental and estimated sensitivities) from higher to lower sensitivity values.
  - Check if the location corresponding to the highest sensitivity is an experimental point or an estimated point. If it is an estimated point, generate the experimental sensitivity for this grid location.
  - If the highest sensitivity point is an experimental point, check the second highest point. If the second highest point is an estimated point, generate the experimental value for this grid location.
  - If the second highest point is also an experimental point, generate the gradient from the second highest point based on the mapped surface. If the upward path from the second highest point leads up to the highest point, label both the points as 1, which implies that they belong to the same hill. Otherwise, label the highest point as 1 and label the second highest point as 2, which indicates that they belong to different hills.
  - Repeat this procedure till an estimated point is located. Meanwhile, keep labeling the experimental points with respect to the hill they belong to and the order of the point on the hill (ex: 3rd highest point in hill 2, etc.).
  - If a hill's highest *ξ* points are experimental points, then label the hill as discovered, which indicates that we have enough information on this hill and collecting information on other hills might be more beneficial.
  - If the search continues till 1% of grid points without finding a suitable candidate for experimentation, halt the search. Locate all the considered points that are inside a sphere of volume 1/500 of the whole search space with center being the highest sensitivity point. Assign a value of “0” for the sensitivities of all points inside this sphere. (Maintain the record of their actual values in another place). Then go to the beginning of Step 2.
5. *Path b* (Diverse Search)
  - The aim of the diverse search is to explore the space to locate new possible candidate hills that were not discovered in the previous searches.
  - Assume that the surface generated by the experimental and estimated points is a probability distribution function (PDF).
  - Generate points by sampling this distribution. For the generation process, we use the Gibbs sampling algorithm. The number of points generated by the Gibbs algorithm is termed as* Number of points to generate the Gibbs sampling*. Since the points are generated from sampling the PDF, the points are denser around the hills and less dense at locations where the efficacy estimate is close to 0.
  - Randomly select one of the generated points as the candidate point and generate its sensitivity experimentally.

### 4.2. Sensitivity Surface Estimation Algorithm

The sensitivity surface estimation algorithm used for our approach is established on the *n* dimensional application of the penalized least square regression analysis based on discrete cosine transform (LS-DTC) proposed by Garcia [21, 22]. The code is generated to compute missing values in data sets. The estimation algorithm contains a parameter *s*, termed as* smoothing parameter* that determines the smoothness of the output. For our case, the smoothness parameter is adjusted to a small value so that the result of surface estimation goes through the actual experimental points. The core of the algorithm is based on minimizing the equation *F*(*y*) = *wRSS* + *s*∗*P*(*y*), where wRSS corresponds to weighted residual sum of squares and *P* is the penalty function related to the smoothness of the output. wRSS can be written explicitly as ${||W^{1/2}\cdot (\hat{y}-y)||}^{2}$, where *y* represents the actual data with missing values and $\hat{y}$ provides the estimate of the data without any missing values. *W* is a diagonal matrix, whose entries represent the reliability of the points and can take values between 0 and 1. For our case, the missing values represent unknown points and are assigned a value of 0 and the experimental points are reliable points which are assigned a value of 1. The solution to $\hat{y}$ that minimizes *F*(*y*) can be generated based on an iterative process starting from an arbitrary initial point ${\hat{y}}_{0}$.

### 4.3. Choice of Parameters

The implementation of the proposed algorithm includes several parameters that can affect the performance of the search process. In this subsection, we present the guiding principles behind the selection of the parameters based on the dimensionality and the total number of grid points in the search space.

#### 4.3.1. Latin Hypercube Numbers (LHNs)

Denote the number of points that will be tested in Step 1 of the algorithm. These points are supposed to provide an initial estimate of the search space. Based on simulations and theoretical analysis, we observe that increasing the number of LHNs provides limited benefit in terms of reaching the maximum sensitivity combination after a certain point. On the other hand, keeping this number too low will cause the program to start the second step with limited knowledge and to search low sensitivity locations. Thus, there is an optimum number of Latin hypercube numbers to maximize the benefit of the algorithm. Although this optimum number depends on the search space; our simulations for 4 surfaces with two different LHNs (10 and 40) illustrate that the proposed algorithm provides better results than ARU algorithm for a fairly large interval of LHNs.

#### 4.3.2. Latin Hypercube Iterations

The Latin hypercube numbers are distributed homogeneously through an iterative algorithm. The iterations maximize the minimum distance between the points. It is desirable to have a higher number of iterations, but after a point, the benefits of increasing the iterations become negligible. For our simulations, we selected a threshold point following which the increase in the maximum minimum distance is negligible.

#### 4.3.3. Number of Iterations to Generate the Sensitivity Surface Estimate

This parameter is related to sensitivity surface estimation algorithm and describes the number of iterations used to find a smooth surface passing through the given points in high dimensional space. A higher value for this parameter will provide a smoothed surface (that still passes through the experimental points) but will carry a high computational time cost. Furthermore, the benefits of increasing the iterations become negligible after a threshold and the output surface becomes more stable. For our examples, we have fixed this number to 100.

#### 4.3.4. Probability of Focused Search

Denote the probability that the search algorithm follows *path* *a*. *Path* *a* attempts to discover the exact local maximum of a hill, and *path* *b* attempts to learn new hills. For all our simulations, this parameter has been assigned a value of 0.3.

#### 4.3.5. Power Used by Inputs

This parameter attempts to emphasize the hills. After normalizing the experimental points, we take the (*n* − 1)th power of the values so that the high peaks are emphasized as compared to dips or grid points with average values in the estimated surface. Thus, the probability of point selection around hills is increased during the Gibbs sampling process.

#### 4.3.6. Number of Points Generated by the Gibbs Sampling

This parameter describes the number of points generated by the Gibbs sampling in *path* *b* of Step 2 of the algorithm. More points provide a better representation of the estimated surface. After a level, the number of points is sufficient to represent the probability distribution and the benefits of increasing the iterations become negligible. We achieve better sampling by increasing this parameter. This parameter is required to be large for problems in higher dimensions or problems containing a huge number of grid points. For our examples, if the number of grid points is below 7500, this parameter has been assigned a value equal to twice the number of grid points. Otherwise, we have fixed the number of the Gibbs sampling points to 15,000.

#### 4.3.7. Clustering Related Parameters

The clustering concept is introduced to avoid the search being stuck in one dominant hill.

#### 4.3.8. Cluster Threshold *ξ*

This denotes the maximum number of experimental points in an individual hill. Further exploration of the hill is paused once this value is reached. For our examples, if the number of drugs (dimensions) *n* is less than 5, *ξ* is assigned a value of 2*n* − 1. Otherwise, the parameter is fixed at 7.

#### 4.3.9. Cluster Break

This parameter denotes the maximum number of high efficacy point estimates in a single hill. If this condition is reached, we assign a value of 0 sensitivity for points around the known top of the hill. This parameter is considered to be around 1% of the total grid points.

#### 4.3.10. Cluster Distance

This parameter represents the radius of the sphere around the hill top for which any grid point within the sphere is assigned a value of 0. The Cluster Distance is selected such that the volume of the sphere is 0.2% of the total volume. The parameter considers that the algorithm has no knowledge of the hills that are narrower than the 0.2% of the total search space.

## Supplementary Material

The supplementary material includes detailed tables for each of the nine simulation experiments conducted to search for the optimized drug concentrations. The supplementary material also includes two tables containing the experimental data for the biological examples.

## Figures and Tables

**Table 1:** Summary of the results for seven synthetic and two experiment based examples. The table illustrates that the cost for our proposed algorithm is significantly lower than the ARU algorithm [15] which has been shown to be efficient than other existing algorithms. Furthermore, the success rate for our algorithm is also better than the ARU algorithm.

	Number of points with ≥0.95 × *Max*⁡_efficacy_	Search grid size	ARU [15] cost	ARU [15] Success Rate	DSS cost	DSS STD	DSS worst case	DSS Success Rate	Initial LHC points
Synthetic examples									
2 DeJong	2	21^2^	46.2	99%	16.0	7.99	48	100%	5
3a	4	11^3^	74	100%	24.7	11.73	72	100%	10
3b	1	11^3^	79.4	100%	52.9	32.20	149	100%	10
4a	1	11^4^	136.8	100%	65.3	14.11	106	100%	40
4a	1	11^4^	136.8	100%	50.7	21.81	159	100%	10
4b	12	11^4^	91.6	100%	52.7	8.80	85	100%	40
4b	12	11^4^	91.6	100%	28.3	9.17	57	100%	10
5a	4	11^5^	80.6	100%	79.3	23.25	157	100%	40
5a	4	11^5^	80.6	100%	61.8	27.58	176	100%	10
5b	8	11^5^	216.8	100%	159.5	90.51	402	100%	40
5b	8	11^5^	216.8	100%	194.2	150.15	647	100%	10

Experiment based examples									
Bacterial inhibition [10]	34	9^2^	4.8	100%	1.85	0.78	3	100%	3
Lung cancer [16]	7	10^2^	12.4	98%	5.97	4.74	23	100%	3

**Table 2:** Expectation and variance of the minimum distances from the optimal point for various values of *n*, *T*, and *m*.

* n *	* T *	* m *	Mean	Variance
5	5	20	4.15	1.77
5	5	40	3.42	1.29
5	5	60	3.04	1.09
10	5	20	11.35	4.29
10	5	40	10.20	3.32
10	5	60	9.59	2.90
15	5	20	19.16	6.88
15	5	40	17.69	5.43
15	5	60	16.92	4.79
5	10	20	8.15	5.25
5	10	40	6.86	3.71
5	10	60	6.21	3.06
10	10	20	21.75	13.35
10	10	40	19.70	10.21
10	10	60	18.62	8.83
15	10	20	36.45	21.76
15	10	40	33.83	17.02
15	10	60	32.45	14.94
