# Supplementary material for: A Diverse Stochastic Search Algorithm for Combination Therapeutics
Source: Biomed Res Int. 2014 Mar 12;2014:873436. doi: 10.1155/2014/873436 (PMC3971504; doi:10.1155/2014/873436)
Supplement: Supplementary file 1 — The supplementary material includes detailed tables for each of the nine simulation experiments conducted to search for the optimized drug concentrations. The supplementary material also includes two tables containing the experimental data for the biological examples. [file 873436.f1.pdf]

A Diverse Stochastic Search Algorithm for Combination Therapeutics -Supplementary material

Mehmet Umut Caglar[1,2] and Ranadip Pal[2*]

[1] Department of Physics, Texas Tech University, Lubbock, TX, USA
[2] Department of Electrical and Computer Engineering, Texas Tech University, Lubbock, TX, USA

Email: Ranadip Pal - ranadip.pal@ttu.edu;

*Corresponding author

Supplementary Material

The supplementary materials include detailed tables of each computational experiment in order to find the optimized drug concentrations.

Supplementary Tables

Table 1: Results for Synthetic Example 1 based on the 2D De Jong function (same distribution as example 2 in [1]). The explanation for the parameters is included in the methods section. The results show the effectiveness of our proposed approach as the average number of iterations required for our approach (cost) is 1/3rd of the ARU [1, 2] approach.

Name of the function	De Jong
Dimension of the problem	2
Interval Min	[-2 -2]
Interval Max	[2 2]
Number of Grid Points	[21×21]
Latin Hypercube Iterations	1000
Number of Iterations for surface estimation	100
Probability of fast search	0.3
Power used for the inputs	1
Number of points to generate Gibbs sampling	800
Number of Repeats	100
Number of Initial (Latin Hyper Cube) Numbers	5
CLUSTERING RELATED PARAMETERS	
Cluster Threshold ξ	3
Cluster Break	10
Cluster Distance	0.447
equation used to simulate experimental results: $z = (1 - x1)^2 + 100 * (x2 - x1^2)^2$	
Number of points with $\geq 0.95\times$ $\text{Max}_{efficacy}$	2 (out of 21^2)
**Proposed algorithm *cost***	**15.96**
**ARU algorithm [2] *cost***	**46.20**
Standard deviation in hundred runs of DSS	7.99
Difference between means of ARU and DSS in terms of std of DSS	3.79σ
Number of iterations in the worst case in DSS	$5 + 43 = 48$
Success Rate for proposed algorithm	100%
Success Rate for ARU	99%

Table 2: Results for Synthetic Example 2. The surface is described by a 3 dimensional response function $z = x1^2 * (sin(x2))^2 * (cos(x3))^2$. The average cost of our algorithm is 33% of the ARU algorithm, illustrating its effectiveness.

name of the function	f3a
Dimension of the problem	3
Interval Min	[-2.5 -2.5 -2.5]
Interval Max	[2.5 2.5 2.5]
Number of Grid Points	[$11 \times 11 \times 11$]
Latin Hypercube Iterations	1000
Number of Iterations for surface estimation	100
Probability of fast search	0.3
Power used for the inputs	2
Number of points to generate Gibbs sampling	15000
Number of Repeats	100
Number of Initial (Latin Hyper Cube) Numbers	10
CLUSTERING RELATED PARAMETERS	
Cluster Threshold ξ	5
Cluster Break	100
Cluster Distance	1.26
equation used to simulate experimental results: $z = x1^2 * (sin(x2))^2 * (cos(x3))^2$	
Number of points with $\geq 0.95\times$ $\text{Max}_{efficacy}$	4 (out of 11^3)
**Proposed algorithm *cost***	**24.7**
**ARU algorithm [2] *cost***	**74.0**
Standard deviation in hundred runs of DSS	11.73
Difference between means of ARU and DSS in terms of std of DSS	4.20σ
Number of iterations in the worst case in DSS	$10 + 62 = 72$
Success Rate for proposed algorithm	100%
Success Rate for ARU	100%

Table 3: Results for Synthetic Example 3. The surface is described by the 3 dimensional response function $z = x3 * peaks(x1, x2)$; where the peaks function is $z = 3*(1-x1)^2 * exp(-(x1^2) - (x2+1)^2) - 10*(x1/5 - x1^3 - x2^5) * exp(-x1^2 - x2^2) - 1/3 * exp(-(x1+1)^2 - x2^2)$ as defined in MATLAB. The average cost of our algorithm is 66.5% of the ARU algorithm, illustrating its effectiveness.

name of the function	f3b
Dimension of the problem	3
Interval Min	[-3 -3 -3]
Interval Max	[3 3 3]
Number of Grid Points	$[11 \times 11 \times 11]$
Latin Hypercube Iterations	1000
Number of Iterations for surface estimation	100
Probability of doing fast search	0.3
Power used for the inputs	2
Number of points to generate Gibbs sampling	15000
Number of Repeats	100
Number of Initial (Latin HyperCube) Numbers	10
CLUSTERING RELATED PARAMETERS	
Cluster Threshold ξ	5
Cluster Break	100
Cluster Distance	1.2599
equation used to simulate experimental results: $z = x3 * peaks(x1, x2)$	
Number of points with $\geq 0.95\times$ $\text{Max}_{efficacy}$	1 (out of 11^3)
**Proposed algorithm *cost***	**52.7**
**ARU algorithm [2] *cost***	**79.4**
Standard deviation in hundred runs of DSS	32.20
Difference between means of ARU and DSS in terms of std of DSS	0.82σ
Number of iterations in the worst case in DSS	$10 + 139 = 149$
Success Rate for proposed algorithm	100%
Success Rate for ARU	100%

Table 4: Results for Synthetic Example 4. The surface is described by the 4 dimensional response function $z = x1 * exp(-(x1^2 + x2^2 + x3^2 + x4^2))$. We run our simulation for two different number of Initial (Latin Hyper Cube) Numbers 40 & 10. The average cost of our algorithm is 47.7% and 37.1% of the ARU algorithm respectively, illustrating its effectiveness.

name of the function	f4a
Dimension of the problem	4
Interval Min	[-2 -2 -3 -3]
Interval Max	[2 2 3 3]
Number of Grid Points	$[11 \times 11 \times 11 \times 11]$
Latin Hypercube Iterations	1000
Number of Iterations for surface estimation	100
Probability of fast search	0.3
Power used for the inputs	3
Number of points to generate Gibbs sampling	15000
Number of Repeats	100
Number of Initial (Latin Hyper Cube) Numbers	40 and 10 for two different tries
CLUSTERING RELATED PARAMETERS	
Cluster Threshold ξ	7
Cluster Break	100
Cluster Distance	2.1147
equation used to simulate experimental results:<br>$z = x1 * exp(-(x1^2 + x2^2 + x3^2 + x4^2))$	
Number of points with $\geq 0.95\times$ Max$_{efficacy}$	1 (out of 11^4)
**Proposed algorithm *cost for run 1 and run 2***	**65.3 and 50.72 respectively**
**ARU algorithm [2] *cost***	**136.8**
Standard deviation in hundred runs of DSS	14.11 and 21.80 respectively
Difference between means of ARU and DSS in terms of std of DSS	5.07σ and 3.95σ respectively
Number of iterations in the worst case in DSS	$40 + 66 = 106$ and $10 + 149 = 159$ respectively
Success Rate for proposed algorithm	100%
Success Rate for ARU	100%

Table 5: Results for Synthetic Example 5. The surface is described by the 4 dimensional response function $z = cos(03 * x1)^2 * sin(03 * x2) * tan(01 * x3) * x4$. We run our simulation for two different number of Initial (Latin Hyper Cube) Numbers 40 & 10. The average cost of our algorithm is 57.5% and 30.9% of the ARU algorithm respectively, illustrating its effectiveness.

name of the function	f4b
Dimension of the problem	4
Interval Min	[-3 -3 -3 -3]
Interval Max	[3 3 3 3]
Number of Grid Points	$[11 \times 11 \times 11 \times 11]$
Latin Hypercube Iterations	1000
Number of Iterations for surface estimation	100
Probability of doing fast search	0.3
Power used for the inputs	3
Number of points to generate Gibbs sampling	15000
Number of Repeats	100
Number of Initial (Latin Hyper Cube) Numbers	40 and 10 for two different tries
CLUSTERING RELATED PARAMETERS	
Cluster Threshold ξ	7
Cluster Break	100
Cluster Distance	2.1147
equation used to simulate experimental results: $z = cos(03 * x1)^2 * sin(03 * x2) * tan(01 * x3) * x4$	
Number of points with $\geq 0.95\times$ Max$_{efficacy}$	12 (out of 11^4)
**Proposed algorithm *cost***	**52.7 and 28.29 respectively**
**ARU algorithm [2] *cost***	**91.6**
Standard deviation in hundred runs of DSS	8.90 and 9.17 respectively
Difference between means of ARU and DSS in terms of std of DSS	4.42σ and 6.90σ respectively
Number of iterations in the worst case in DSS	$40 + 45 = 85$ and $10 + 47 + 57$ respectively
Success Rate for proposed algorithm	100%
Success Rate for ARU	100%

Table 6: Results for Synthetic Example 6. The surface is described by the 5 dimensional response function $z = exp(-x1) * cos(x2)^2 * x3^2 * (exp(-(x4+2)^2 - (x5+3)^2) + exp(-(x4-2)^2 - (x5-3)^2))$. We run our simulation for two different number of Initial (Latin Hyper Cube) Numbers 40 & 10. The average cost of our algorithm is 98.4% and 76.7% of the ARU algorithm respectively, illustrating its effectiveness.

name of the function	function f5a
Dimension of the problem	5
Interval Min	[-2 -2 -4.5 -4.5 -4.5]
Interval Max	[2 2 4.5 4.5 4.5]
Number of Grid Points	$[11 \times 11 \times 11 \times 11 \times 11]$
latin Hypercube Iterations	1000
Number of Iterations for surface estimation	100
Probability of doing fast search	3
Power used for the inputs	4
Number of points to generate Gibbs sampling	15000
Number of Repeats	100
Number of Initial (Latin HyperCube) Numbers	40 and 10 for two different tries
CLUSTERING RELATED PARAMETERS	
Cluster Threshold ξ	7
Cluster Break	1000
Cluster Distance	2.8854
equation used to simulate experimental results: $z = exp(-x1) * cos(x2)^2 * x3^2 * (exp(-(x4+2)^2 - (x5+3)^2) + exp(-(x4-2)^2 - (x5-3)^2))$	
Number of points with $\geq 0.95\times$ Max$_{efficacy}$	4 (out of 11^5)
**Proposed algorithm *cost***	**79.25 and 61.78 respectively**
**ARU algorithm [2] *cost***	**80.6**
Standard deviation in hundred runs of DSS	23.25 and 27.58 respectively
Difference between means of ARU and DSS in terms of std of DSS	0.06σ and 0.68σ respectively
Number of iterations in the worst case in DSS	$40 + 117 = 157$ and $10 + 166 = 176$ respectively
Success Rate for proposed algorithm	100%
Success Rate for ARU	100%

Table 7: Results for Synthetic Example 7. The surface is described by the 5 dimensional response function $z = 1/2 * peaks(x1, x2) * cos(05 * x3) * sin(05 * x4) * (x5)^2$; where the peaks function is $z = 3 * (1 - x1)^2 * exp(-(x1^2) - (x2 + 1)^2) - 10 * (x1/5 - x1^3 - x2^5) * exp(-x1^2 - x2^2) - 1/3 * exp(-(x1 + 1)^2 - x2^2)$ as defined in MATLAB. We run our simulation for two different number of Initial (Latin Hyper Cube) Numbers 40 & 10. The average cost of our algorithm is 73.5% and 89.6% of the ARU algorithm respectively, illustrating its effectiveness.

name of the function	function 5b
Dimension of the problem	5
Interval Min	[-3 -3 -3 -3 -3]
Interval Max	[3 3 3 3 3]
Number of Grid Points	$[11 \times 11 \times 11 \times 11 \times 11]$
Latin Hypercube Iterations	1000
Number of Iterations for surface estimation	100
Probability of doing fast search	0.3
Power used for the inputs	4
Number of points to generate Gibbs sampling	15000
Number of Repeats	100
Number of Initial (Latin Hyper Cube) Numbers	40 and 10 for two different tries
CLUSTERING RELATED PARAMETERS	
Cluster Threshold ξ	7
Cluster Break	1000
Cluster Distance	2.8854
equation used to simulate experimental results: $z = 1/2 * peaks(x1, x2) * cos(05 * x3) * sin(05 * x4) * (x5)^2$	
Number of points above .95 success rate (after normalization)	8 (out of 11^5)
Number of points with $\geq 0.95\times$ Max$_{efficacy}$	8 (out of 11^5)
**Proposed algorithm *cost***	**159.47 and 194.15 respectively**
**ARU algorithm [2] *cost***	**216.8**
Standard deviation in hundred runs of DSS	90.51 and 150.15 respectively
Difference between means of ARU and DSS in terms of std of DSS	0.63σ and 0.15σ respectively
Number of iterations in the worst case in DSS	$40 + 362 = 402$ and $10 + 637 = 647$ respectively
Success Rate for proposed algorithm	100%
Success Rate for ARU	100%

Table 8: Results for Biological Example 1 related to normalized bacterial (S. aureus) inhibition (2D) response described in [3]. The combination drugs considered are Trimethoprim and Sulfamethoxazole. The average cost of our algorithm is 42% of the ARU algorithm illustrating its effectiveness.

Source of data set	Bacterial inhibition [3]
Dimension of the problem	2
Name of the drugs	[Trimethoprim, Sulfamethoxazole]
Trimethoprim intervals	[0, 0.08, 0.16, 0.32, 0.63, 1.25, 2.5, 5, 10] μM
Sulfamethoxazole intervals	[0, 0.31, 0.62, 1.25,2.5, 5, 10, 20, 40] μM
Number of Grid Points	$[9 \times 9]$
Latin Hypercube Iterations	1000
Number of Iterations for surface estimation	100
Probability of fast search	0.3
Power used for the inputs	1
Number of points to generate Gibbs sampling	200
Number of Repeats	100
Number of Initial (Latin Hyper Cube) Numbers	3
CLUSTERING RELATED PARAMETERS	
Cluster Threshold ξ	3
Cluster Break	10
Cluster Distance	0.44721
Number of points with $\geq 0.95\times$ Max$_{efficacy}$	34 (out of 9^2)
**Proposed algorithm *cost***	**1.85**
**ARU algorithm [2] *cost***	**4.50**
Standard deviation in hundred runs of DSS	0.78
Difference between means of ARU and DSS in terms of std of DSS	3.77σ
Number of iterations in the worst case in DSS	$3 + 0 = 3$
Success Rate for proposed algorithm	100%
Success Rate for ARU	100%

Table 9: Results for Biological Example 2 related to normalized lung cancer inhibition response (2D) described in [4]. The combination drugs considered are Pentamidine and Chlorpromazine The average cost of our algorithm is 48% of the ARU algorithm, illustrating its effectiveness. The success percentage for our algorithm is 100% whereas ARU has a success rate of 98%.

Source of the data	Lung Cancer Response [4]
Dimension of the problem	2
Name of the drugs	[Pentamidine Chlorpromazine]
Pentamidine intervals	[0, 0.25, 0.4, 0.6, 0.8, 1, 1.5, 2,4, 6.8] μM
Chlorpromazine intervals	[0, 1, 2, 4, 6, 8, 12, 16, 20, 22] μM
Number of Grid Points	$[10 \times 10]$
Latin Hypercube Iterations	1000
Number of Iterations for surface estimation	100
Probability of fast search	0.3
Power used for the inputs	1
Number of points to generate Gibbs sampling	100
Number of Repeats	100
Number of Initial (Latin Hyper Cube) Numbers	3
CLUSTERING RELATED PARAMETERS	
Cluster Threshold ξ	3
Cluster Break	10
Cluster Distance	0.447
Number of points with $\geq 0.95\times$ Max$_{efficacy}$	7 (out of 10^2)
**Proposed algorithm *cost***	**5.97**
**ARU algorithm [2] *cost***	**12.40**
Standard deviation in hundred runs of DSS	4.74
Difference between means of ARU and DSS in terms of std of DSS	1.36σ
Number of iterations in the worst case in DSS	$3 + 20 = 23$
Success Rate for proposed algorithm	100%
Success Rate for ARU	98%

Table 10: Bacterial infection data set from [3] Figure 3a

Sulfamethoxazole (μM) \ Trimethoprim (μM)	0.00	0.08	0.16	0.32	0.63	1.25	2.50	5.00	10.00
40.00	60	91	90	94	92	94	92	96	94
20.00	27	91	90	94	92	94	93	96	93
10.00	12	84	88	94	92	95	93	96	95
5.00	8.4	69	84	92	91	94	92	95	94
2.50	5	44	75	78	89	92	91	93	92
1.25	1.7	11	60	78	81	93	89	94	91
0.62	1.8	7.9	17	42	64	88	92	96	94
0.31	1.1	-0.4	8.7	21	39	77	91	96	95
0.00	6.8	-1.7	5.4	6.4	31	44	73	88	94

Table 11: lung cancer inhibition response data set from [4] figure 4a

Chlorpromazine (μM) \ Pentamidine (μM)	0.00	0.25	0.40	0.60	0.80	1.00	1.50	2.00	4.00	6.80
22.00	71	63	71	68	78	75	80	78	82	77
20.00	67	63	69	66	79	75	81	78	83	80
16.00	57	56	62	57	69	66	74	77	80	74
12.00	36	44	57	51	66	62	72	74	75	75
8.00	21	29	40	36	42	37	53	62	71	68
6.00	9	14	31	26	32	39	52	55	66	71
4.00	-12	10	5	14	24	25	35	30	59	68
2.00	-3	-4	15	5	22	19	29	44	59	59
1.00	-12	-11	-4	0	9	0	21	33	56	64
0.00	-3	-9	9	-1	1	-3	18	41	56	59

References

1. Yoon BJ: **Enhanced stochastic optimization algorithm for finding effective multi-target therapeutics**. *BMC Bioinformatics* 2011, **12**(Suppl 1):S18+.
2. Kim M, Yoon BJ: **Adaptive reference update (ARU) algorithm. A stochastic search algorithm for efficient optimization of multi-drug cocktails**. *BMC Genomics* 2012, **13**(Suppl 6):S12.
3. Zimmermann GR, Lehár J, Keith CT: **Multi-target therapeutics: when the whole is greater than the sum of the parts.** *Drug discovery today* 2007, **12**(1-2):34–42.
4. Borisy AA, Elliott PJ, Hurst NW, Lee MS, Lehár J, Price ER, Serbedzija G, Zimmermann GR, Foley MA, Stockwell BR, et al.: **Systematic discovery of multicomponent therapeutics**. *Proceedings of the National Academy of Sciences* 2003, **100**(13):7977–7982.
